# KnowTox: pipeline and case study for confident prediction of potential toxic effects of compounds in early phases of development

**DOI:** 10.1186/s13321-020-00422-x

**Published:** 2020-04-14

**Authors:** Andrea Morger, Miriam Mathea, Janosch H. Achenbach, Antje Wolf, Roland Buesen, Klaus-Juergen Schleifer, Robert Landsiedel, Andrea Volkamer

**Affiliations:** 1 In Silico Toxicology and Structural Bioinformatics, Institute of Physiology, Charité Universitätsmedizin Berlin, Charitéplatz 1, Berlin, Germany; 2 BASF SE, 67056 Ludwigshafen, Germany

**Keywords:** Toxicity prediction, ToxCast, Read-across, Random forest, Conformal prediction, Confidence estimation, Applicability domain, Case study, Androgen receptor, Triazoles

## Abstract

Risk assessment of newly synthesised chemicals is a prerequisite for regulatory approval. In this context, in silico methods have great potential to reduce time, cost, and ultimately animal testing as they make use of the ever-growing amount of available toxicity data. Here, KnowTox is presented, a novel pipeline that combines three different in silico toxicology approaches to allow for confident prediction of potentially toxic effects of query compounds, i.e. machine learning models for 88 endpoints, alerts for 919 toxic substructures, and computational support for read-across. It is mainly based on the ToxCast dataset, containing after preprocessing a sparse matrix of 7912 compounds tested against 985 endpoints. When applying machine learning models, applicability and reliability of predictions for new chemicals are of utmost importance. Therefore, first, the conformal prediction technique was deployed, comprising an additional calibration step and per definition creating internally valid predictors at a given significance level. Second, to further improve validity and information efficiency, two adaptations are suggested, exemplified at the androgen receptor antagonism endpoint. An absolute increase in validity of 23% on the in-house dataset of 534 compounds could be achieved by introducing KNNRegressor normalisation. This increase in validity comes at the cost of efficiency, which could again be improved by 20% for the initial ToxCast model by balancing the dataset during model training. Finally, the value of the developed pipeline for risk assessment is discussed using two in-house triazole molecules. Compared to a single toxicity prediction method, complementing the outputs of different approaches can have a higher impact on guiding toxicity testing and de-selecting most likely harmful development-candidate compounds early in the development process.

## Introduction

Before newly developed chemicals can be approved, their potential toxic effects on humans and the environment inevitably need to be assessed. Most regulations such as REACH [1] require animal studies for risk assessment. E.g. more than 540,000 animals were employed in Germany in 2017 for production, quality control, and safety assessment [2].

Given the ever growing amount of available toxicity data, computational toxicity prediction methods have great potential to reduce time, cost, and ultimately animal testing. Using historical data, they can help to disclose relationships between compounds that would not have been identified manually and, thus, reveal potential risk of compounds in early phases of development. In silico predictions can hint at potentially hazardous interactions or critical structural moieties of new molecules. If the corresponding assays are conducted first, harmful compounds can be filtered out before performing a wide range of additional experiments. Moreover, in silico methods can support product optimisation and reduce long-term animal toxicity studies [3, 4].

In silico strategies for supporting risk assessment range from computational read-across approaches and search for substructural alerts to statistical methods. Especially, quantitative structure-activity relationship (QSAR) techniques such as machine learning (ML) [5] methods require a large precompiled dataset.

The US Environmental Protection Agency (EPA) has provided the ToxCast dataset [6] consisting of roughly 8000 compounds, such as pharmaceuticals, pesticides, and environmental chemicals, that were tested on up to 1000 endpoints, e.g. cell cycle, steroid receptors, and cytotoxicity. ToxCast has since been used: to develop QSAR models [7–9]; to generate biological fingerprints for in vivo endpoint predictions [10]; to decipher adverse outcome pathways [7, 11]; and as a basis for read-across [12–14].

Read-across is a common, often manual, approach in toxicology [12, 15, 16], based on the assumption that similar molecules can evoke similar toxic effects. Missing information on query chemicals’ properties may be gathered by reading across information from very similar molecules. Using different molecular encodings and diverse similarity measures, computers can search through large compound databases to identify the most similar compounds and—given a decent similarity—transfer knowledge to a query compound. Prerequisite for successful read-across is a robust and reproducible test system of the underlying experimental data [16], i.e. a standardised assay set-up to ensure comparable read-outs. Another challenge is the determination of the amount of required similarity between two compounds that allows safe and reliable knowledge transfer.

Since often not the complete molecule, but rather a specific functional group or fragment, is responsible for an unwanted effect, identifying such toxic substructures in a query molecule is of high practical value. Several authors published lists of toxic alerts or other undesired substructures which can be used to flag novel compounds [17, 18]. For instance, the OCHEM ToxAlert server allows to browse and query structural alerts for various toxicological endpoints [17, 19].

Often the relationship between molecular structure and toxic effect is not linear, thus, statistical methods such as QSAR models are applied to recognise more complex patterns in datasets. The set-up of high-performing toxicity prediction models has recently been promoted in the Tox21 Data Challenge. Research groups competed in model performance on 12 nuclear receptor and stress response pathways trained on roughly 10,000 compounds [20], including various ML algorithms such as random forest, support vector machine, and deep learning approaches [21–23]. The winning models on all 12 endpoints showed AUC-ROC scores between 0.81 and 0.95 on an external blinded test set [20].

Fuart Gatnik and Worth published an overview on publicly and commercially available software tools, such as the well-known TOPKAT [24] and DEREK [25] methods, for toxicity prediction [26]. Concluding, the authors stated that the availability and quality of the models is endpoint-dependent and they emphasised on the observation that generally more research is needed in terms of assessment of the applicability of the in silico models. Besides pure predictions, for practical applications, knowledge about the applicability domain, i.e. the space of chemicals the model can make reliable predictions for, is of major importance. Hanser et al. [27] suggested to further divide this concept into three domains: applicability, reliability, and decidability. The applicability domain indicates whether a model can be applied to make a prediction for a certain use case. It can be defined, for example, by a convex hull around the main components of a principal component analysis (PCA) fitted on the features of the training data. The reliability domain gives information on whether the obtained prediction is reliable enough for the use case. It can be explored by investigating the average distance to the nearest neighbours. The decidability domain returns if a clear decision can be made, based on the outcome of the prediction. Therefore, the distribution of the nearest neighbour’s labels can be analysed [27].

A recently promoted method for confidence estimation, especially regarding reliability and decidability, is conformal prediction (CP) [28, 29]. A conformal predictor returns, whether enough evidence is given to reliably assign the query substance to a certain class. CP models have recently been developed and applied in drug discovery [29–31], and toxicology, e.g. to predict cytoxicity [32], endocrine disruption [33], and skin penetration [34]. Moreover, recently, eMolTox was introduced, a webserver offering 174 CP models [35]. However, to the best of our knowledge, few information about applying such models to real-world use cases has been published.

In this work, KnowTox, a holistic toxicity prediction approach, that integrates refined conformal predictors, structural alerts, and read-across support based on molecular similarity, is introduced and applied to industrial chemicals. The main source of toxicity information is the publicly available ToxCast dataset. Being aware of the challenge to apply ML models trained on public data to an industrial setting, first, the CP model performance was optimised focusing on the androgen receptor endpoint and validated on an in-house dataset. The focus is on endocrine disruption as a disturbance of steroidal hormone homeostasis can cause severe toxic effects, e.g. leading to male feminisation or reproduction disorders [36, 37]. Thus, screening for agonistic and antagonistic activities on androgen and estrogen receptors is frequently conducted in yeast cells (so-called YES- and YAS assays [38]) and sufficient validation data is available. Finally, CP models were trained using the same CP set-up for another 87 ToxCast endpoints with enough training data available. Moreover, with KnowTox, the refinement of chemical structures is guided by the implementation of warnings about unfavourable structural moieties described in literature [35, 39, 40]. To support read-across, a similarity search is proposed which can automatically point to toxic effects in cells and interactions known for the most similar molecules within ToxCast. In a case study, the potential of KnowTox is exemplified on two in-house triazoles. Multiple components of the KnowTox pipeline indicated liver toxicity and endocrine disruption which is in accordance with literature and retrospective test results.

## Data and methods

In the following, first the main datasets and their preparation will be introduced, followed by the individual methods for the KnowTox toxicity prediction tool, including CP, PCA, toxic substructure and similarity search.

### Datasets

#### ToxCast dataset

The source of molecules and assay data for KnowTox is the freely available ToxCast dataset provided by the EPA. It consists of over 8000 compounds tested on up to 1092 different toxic endpoints. The data was downloaded from EPA's National Center for Computational Toxicology [41] (date 23.06.2017). Toxicity values were directly adopted from the hitcalls defined by the EPA. Flags were not considered, but endpoints corresponding to background measurements were excluded. This yielded a sparse matrix of 8390 compounds with respective toxicity value (0,1, NaN) per tested endpoint (985 total). The ToxCast dataset represented the basis for the similarity search as well as for CP.

#### Androgen receptor datasets

To validate and optimise the CP set-up for model application on external data, three datasets for androgen receptor antagonism (AA) were collected (see Table 1).

**Table 1:** Size and purpose of androgen receptor antagonism datasets used to validate the original conformal prediction model

Dataset	Purpose	Actives	Inactives
ToxCast-AA 762	Train and test model	868	5842
In-house-AA	Validation I	280	254
External-AA	Validation II	160	201

##### ToxCast-AA

The AA assay from ToxCast (assay endpoint id 762) was selected. The assay originates from the Tox21 platform and was conducted in human kidney cells (HEK293T). It is a reporter gene assay that measures beta lactamase induction upon antagonistic activity regulated by the human androgen receptor. Activity data are available for 6710 chemicals.

##### In-house-AA

The in-house dataset from BASF consists of 534 chemicals tested in YES/YAS assays [38]. They are mainly pesticides, such as fungicides and herbicides, and not part of the ToxCast dataset. These compounds were not launched on the market but failed for different reasons during the development. In the YAS assay, human androgen receptor is expressed in yeast cells. Upon binding of an androgenic compound, the lacZ reporter gene is activated, which is responsible for expression of $\beta$-galactosidase. Presence of this enzyme can be detected by a colour change. Anti-androgenic effects can be observed if binding of a known androgenic agent is inhibited and thus the colour change is reduced or does not occur at all. YES assays are conducted similarly, but in yeast cells that express the human estrogen receptor.

##### External-AA

Another external dataset, collected by Jensen et al. [42] and by Vinggaard et al. [43] for QSAR modelling, was downloaded from Norinder et al. [33]. The dataset consists of initially 925 molecules that were especially selected to represent a large chemical space [43]. 361 of these molecules, that are not part of ToxCast, were used in this study. Data originate from an AA assay reporting luminescence response upon inhibition of androgen binding to a synthetic androgen receptor and following gene expression in chinese hamster ovary cells.

### Dataset preprocessing

#### Standardisation

**Fig. 1:**
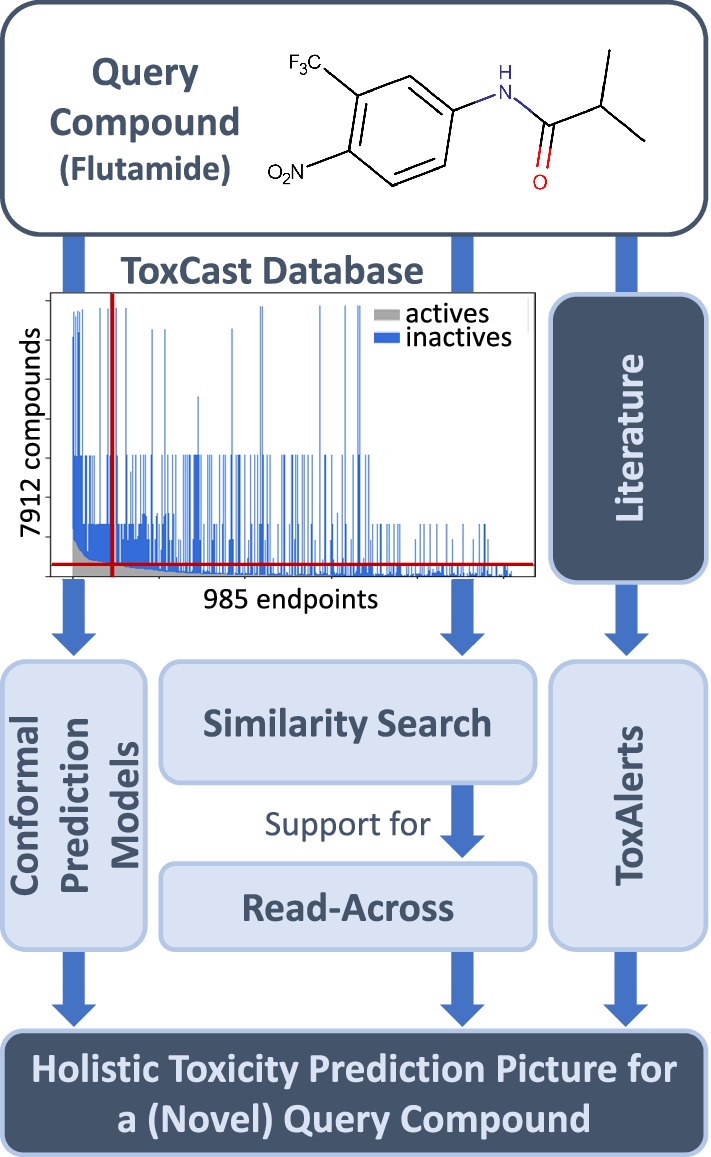
Overview of KnowTox. Combining toxicity information from different sources, the complementary outputs of the KnowTox tool help to generate a holistic toxicity prediction picture for a novel query compound. ToxCast Database bar plot: Number of actives (grey) and inactives (blue) available per endpoint, sorted by number of actives. Red vertical line: CP models were built for the endpoints on the left side of the threshold line (at least 300 active and inactive compounds tested, red horizontal line)

Each molecule was standardised by applying the following workflow: first, duplicates (compounds tested more than once for a specific endpoint) were removed. Only one instance was kept if the assay outcomes agreed—otherwise both instances were discarded. Next, molecules were standardised using the IMI eTox project standardiser tool [44]. This included discarding non-organic compounds, application of certain structure standardisation rules (e.g. handling of tautomers, shifting protons between heteroatoms), neutralisation, and removal of, mainly organic, salts. Due to this standardisation step new duplicates occurred; they were treated as described above. Next, remaining mixtures as well as fragments with less than three heavy atoms were removed yielding a cleaned dataset of 7912 ToxCast molecules tested on up to 985 endpoints (see Fig. 1, top). The resulting total number of active and inactive compounds for the AA datasets are listed in Table 1.

#### Descriptor calculation

For similarity search as well as CP, all molecules were encoded by molecular descriptors implemented in RDKit. For similarity search and the original CP model, a combination of the SMARTS-pattern based MACCS keys and the circular-environment based Morgan fingerprint (radius 3, 1024 bits) was chosen. MACCS keys [45] represent the presence or absence of predefined functional groups. Morgan fingerprints [46] are a more abstract representation of a molecule, covering every atom and its circular environment including all atoms and bonds within a defined radius. Concatenation of the two descriptors resulted in a 1191-bit long feature vector representation per molecule. For the normalised and normalised + balanced CP models (see Table 2), the concatenated descriptor (binary values) was reduced to bits with feature variance of equal or higher than 0.01. Additionally, 200 physicochemical descriptors within RDKit [47] (float values) were calculated, normalised and reduced (feature variance threshold 0.001). Finally, these two descriptor sets were concatenated resulting in a feature vector of length 1341. Normalisation of physicochemical parameters and feature reduction were performed based on all standardised ToxCast molecules.

**Table 2:** Conformal prediction models built for androgen receptor antagonism

Model name	Descriptors	nc^a^	Balancing
Original	Morgan + MACCS	Default	No
Normalised	Morgan + MACCS + physchem^b^	Normalised	No
Normalised + balanced	Morgan + MACCS + physchem^b^	Normalised	Yes

^a^nc: nonconformity score

^b^physicochemical descriptors

### KnowTox pipeline

KnowTox allows input of a query molecule and offers in silico support for risk assessment from various view points, comprising CP, similarity search to support read-across, and search for toxic substructures (see Fig. 1). In the following, the individual methods will be explained.

#### Machine learning and conformal prediction

##### General CP workflow

**Fig. 2:**
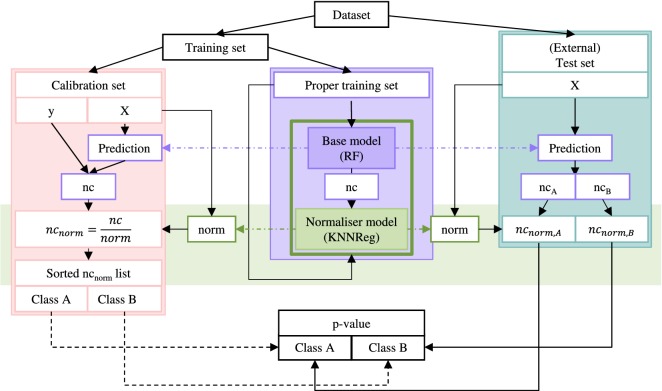
Schematic description of CP workflow. Data is split into training and test set (blue box). The training set is further divided into calibration (red box) and proper training set (violet box). An ML model is fitted on the proper training set and used to predict compounds of the calibration and test set. Predictions are transformed into nonconformity scores (nc scores). Calibration is conducted by sorting the nc scores of the calibration set (class-wise, mondrian) into two lists. The nc score of a test compound is arranged in the list and thus the p-value calculated. An additional normaliser model (green box) can optionally be fitted on the descriptors and nc scores of the compounds of the proper training set

The CP framework is built on top of ML models and is designed to make valid predictions at a given significance level (SL), assuming exchangeability [30]. An overview of the CP workflow used here (offline-mode, binary classification setting) is shown in Fig. 2. Similar to the standard ML setting, the dataset is stratified and randomly split into a training and a test set. Then, an additional calibration step is introduced, in which training data is further split into a proper training and a calibration set. An underlying ML model, e.g. a random forest, is fitted on the proper training set and used to make a prediction (probability $\hat{p}$) for compounds of the calibration and the test set. The prediction outcome per class is transformed into a so-called nonconformity score (nc score). A nonconformity error function is chosen in the way that more ideal predictions yield lower nc scores; a typical error function for random forest classification models is the inverse probability (Eq. 1):

1 $$\begin{aligned} nc\;score = 1 - \hat{p} \end{aligned}$$

To improve reliability estimation of predictions, an additional normaliser regression model (e.g. kNN) can be fitted on the descriptors of the proper training set and their nc scores. For a new compound, the normaliser regression model returns a normalised nc score ($\hbox {nc score}_{norm}$), by dividing the nc score of the compound by the average nc score of the compound’s k nearest neighbours within the proper training set (see Eq. 2).

2 $$\begin{aligned} nc \;score_{norm}=\frac{nc\;score}{norm} \end{aligned}$$

Using mondrian classification [48], the CP algorithm generates for each class a sorted list of nc scores or $\hbox {nc scores}_{norm}$ for the calibration set. The ratio of these nonconformity scores higher, and thus more nonconforming, than the nc score predicted for a query compound is called p-value. If a p-value is larger than a given SL $\epsilon$ (maximum allowed error rate), that label is assinged to a compound. Thus, for a binary classification problem, the output prediction set per compound contains either one class ({0},{1}), both classes ({0,1}), or an empty prediction set ({}). To obtain more stable predictions, multiple conformal predictors can be trained and the p-values are averaged, so-called aggregated conformal predictors (ACPs) [49] are generated.

CPs are typically evaluated regarding validity, efficiency and accuracy. Validity is defined as the ratio of predictions containing the correct label. A common efficiency measure is the ratio of single class predictions (SCPs). Accuracy of SCPs corresponds to the ratio of correct SCPs divided by all SCPs.

##### CP model set-up in this study

Three different settings for CP were applied. The corresponding models will further be called ’original’, ’normalised’ and ’normalised + balanced’ model (see Table 2).

For the original model, data was split into 80% training and 20% test data. Within each loop of a fivefold cross-validation, an ACP with 25 loops was generated. In each ACP loop, training data was split into 70% proper training and 30% calibration data (see Carlsson et al. [49]). Random forest models (500 estimators, else default parameters) were trained on the proper training sets and the predictions calibrated using the respective calibration sets (inverse probability error function, mondrian condition). P-values were aggregated by their median as suggested by Linusson et al. [50]. Finally, the mean p-value of the cross-validation was calculated.

For the normalised model, information from the nearest neighbours in the training set was taken into account as described in Eq. 2. The normaliser model was fitted using the KNNRegressor algorithm (scikit-learn, default parameters).

In the normalised + balanced model, per ACP loop, the proper training and calibration data were five times randomly subsampled to equal numbers of actives and inactives.

After evaluation, normalised + balanced models were built for all ToxCast endpoints for which at least 600 compounds were measured—300 active (toxic) and 300 inactive (non-toxic)—yielding 88 CP models (see Fig. 1, ToxCast Database bar plot, vertical red threshold line).

#### Principal component analysis (PCA) for AA data

For chemical space analysis, a 2-component PCA was fitted on ToxCast AA data. ToxCast-AA, in-house-AA, and external-AA data were projected into the descriptor space. Same descriptors were used as described for the normalised and normalised + balanced CP models.

#### Structural alerts

To identify potentially toxic or unwanted substructures in the query molecules, known structural alerts, encoded as SMARTS patterns, collected from literature are used. A list of 919 structural alerts incorporated in KnowTox was kindly provided by the authors of eMolTox [35]. Using RDKit, a substructure search for all these patterns in the query molecule is performed. Matching substructures are stored together with information about the associated toxic effect, individually highlighted in the molecule and labelled.

#### Similarity search and read-across

Computational support for read-across in KnowTox is implemented via a similarity search and subsequent extraction of information from ToxCast. For similarity search, a query compound is compared to all ToxCast compounds using the calculated descriptors. Finally, ToxCast compounds are ranked by Tanimoto similarity to the query compound. The tool returns the most similar compounds together with their respective maximum common substructure (MCS) with the query compound highlighted. Subsequent read-across is supported by extracting experimental activity of these similar molecules from the ToxCast dataset for all 985 endpoints.

#### Python libraries and versions

Molecules were standardised using the standardiser library [44] version 0.1.9. Descriptor calculation, structural alerts and similarity search were implemented using RDKit [47] version 2018.03.4. For local calculation of feature variances, normalisation of physicochemical parameters, and PCA, scikit-learn [51] version 0.19.2 was used. CP models were trained using nonconformist [52] version 2.1.0 and underlying ML models using scikit-learn (version 0.19.0). Plots were generated using matplotlib version 2.2.3.

#### Supplementary information on github

A github repository with supplementary information is provided under https://github.com/volkamerlab/knowtox_manuscript_SI. It contains the pre-processed ToxCast and external-AA data, as well as a notebook demonstrating the conformal prediction set-ups used in this work.

## Results and discussion

In this section, first, the optimisation of the CP model with respect to applicability to in-house and external data will be discussed, with focus on prediction of AA assay outcome as well as on the complete set of 88 ToxCast endpoints. Finally, the full spectrum of predictions provided by KnowTox will be show cased on two triazoles.

### Conformal predictors—validation of AA model

The aim of this study was to generate reliable toxicity prediction CP models which can be applied to in-house industrial chemicals. Data from the freely available and comparably large ToxCast toxicity database is used, which contains experimental data from consistent measurements per assay endpoint. As there is a shift in chemical and descriptor space expected, when applying the models to in-house compounds, it is important to validate the method carefully. Thus, a CP model to predict androgen receptor antagonism (AA) was selected for validation. Here, an in-house dataset with 534 industrial compounds was available, as well as another external dataset with 361 compounds. AA is an important endpoint to examine a compounds’ risk for endocrine disruption disorders such as male feminisation or sexual disruption in fish [36] and other species [37].

**Fig. 3:**
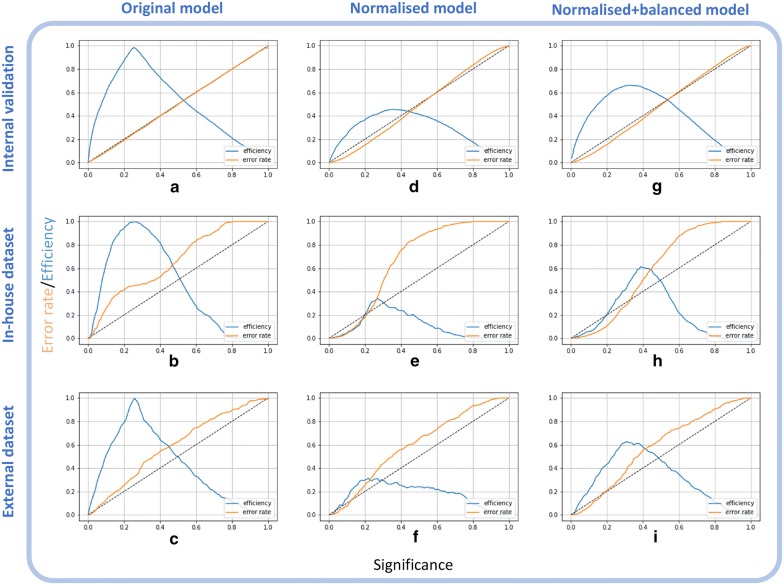
Calibration plots of the original, normalised, and normalised + balanced ToxCast-AA models applied to internal validation, in-house-AA and external-AA data

By design, conformal predictors are valid at a given SL, assuming data exchangeability [30]. This is also observed when training a standard CP model on ToxCast AA data. Figure 3a shows a calibration plot of the internal validation of the original ToxCast-AA model. Ideally, the error rate is equal to the significance (diagonal in Fig. 3a), thus, the original ToxCast-AA model is valid (orange line in Fig. 3a). Also, high efficiency (ratio of SCPs) of 0.87 is achieved at SL 0.2. Since evaluation at SL 0.2 is commonly used in literature, the values will also be given when describing the further validation process. Furthermore, the performance of the ToxCast-AA model is in line with two other AA models extracted from literature (see Table 3), i.e. the eMolTox webserver [35] and work by Norinder et al. [33]. Validity, efficiency, and accuracy values for all three studies (if reported) at SL 0.2 are in the range of 0.76–0.83, 0.87–0.99, and 0.79–0.82, respectively. Although the above described AA models all use CP, they are only partly directly comparable as underlying data, techniques and/or features differ (see Table 4).

**Table 3:** Comparison of original conformal prediction model for androgen receptor antagonism at 0.2 SL with other studies from literature

Model	Validity			Efficiency	Accuracy	
	All	Class 1^c^	Class 0^c^	All	Class 1^c^	Class 0^c^
KnowTox-AA	0.81	0.82	0.81	0.87	0.80	0.78
eMolTox [35]^a^	–	0.76–0.81	0.81–0.82	0.94–0.99	–	–
Norinder et al. [33]^b^	0.80–0.81	0.81–0.83	0.79–0.82	–	0.79–0.82	0.78–0.79

^a^Values of models fitted on two different AA datasets.

^b^Three models with different fingerprints trained on one AA dataset

^c^class 1 = actives, class 0 = inactives

**Table 4:** Information on KnowTox-AA and other CP methods using the random forest ML algorithm to predict androgen receptor antagonism

Method	Data source: actives/inactives	CP aggregation method^a^	Descriptors
KnowTox-AA	ToxCast: 868/5842	ACP	Morgan+MACCS ($+\hbox {physchem}$)
eMolTox [35]^b^	$\hbox {Literature}$:^d^ (1) 532/6207 (2) 406/6256	ACP	$\hbox {Morgan + physchem}$
Norinder et al. [33]^c^	Jensen et al. [42]: 293/637	CCP	(1) Dragon (2) Signatures (3) Physchem

^a^*ACP* aggregated conformal predictor,* CCP* cross-conformal predictor [48]

^b^Two models ((1), (2)) fitted on two different AA datasets

^c^Three models ((1), (2), (3)) with different fingerprints trained on one AA dataset

^d^Data for a total of 174 CP models originated from ChEMBL, Pubchem, Toxnet, eChemPortal databases and literature [35]

Note that some other QSAR models for AA have been published, based on similar data, using random forest, deep learning [53], and the Case Ultra system [54]. Since set-up and reported performance measures differ from this CP study, they can not directly be compared. Very recently, CoMPARA, an extensive study on androgen receptor modelling, was published by Mansouri et al. [55]. Scientists from 25 research groups have contributed to consensus models for androgen receptor binding, agonism, and antagonism with a predictive accuracy of 78% for the AA evaluation set (which is in the same range as the CP accuracy (SCP) obtained for the original KnowTox-AA model, see Table 3). The individual AA models were trained on 1525 ToxCast chemicals using, amongst others, neural networks as well as tree-based and linear modelling approaches.

When applying the original ToxCast-AA model to the libraries of in-house (Fig. 3b) and external molecules (Fig. 3c), validity at 0.2 SL dropped from 0.81 for the internal validation to 0.59 for the in-house dataset. Furthermore, a high discrepancy was observed between the ratio of correct predictions of the active (0.98) and inactive (0.16) class for the in-house data (see Additional file 1: Table S1). Reasons for lower validity could be lacking exchangeability between the compounds of the datasets (pharmaceuticals vs. industrial chemicals) and data originating from different assays.

**Fig. 4:**
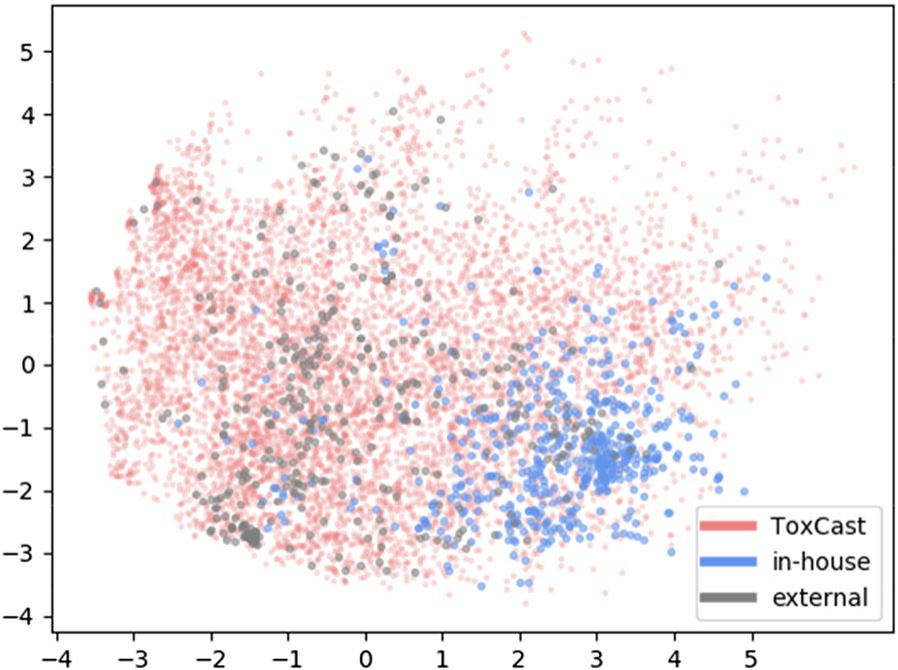
ToxCast, in-house and external data are projected into the descriptor space of a 2-component PCA trained on ToxCast-AA data

Hence, the chemical space was analysed with respect to 1) the most similar compounds and 2) the descriptor space using PCA. First, the average Tanimoto similarity to the ten most similar molecules in ToxCast decreases from 0.51 for intra ToxCast similarity to 0.44 for external data and 0.37 for in-house data. Second, the PCA (Fig. 4) reveals that the in-house data (blue dots) shows the highest density in the lower right corner, which is different from the dense area of the ToxCast data (red dots). The external dataset (grey dots) is more similar to the ToxCast distribution, occupying a dense area in the middle of the plot. Varying distribution and density contribute to poor exchangeability between the different datasets.

To improve reliability of the models, the chemical space was considered by including information about the nearest neighbours to normalise the conformal predictions. While such a normalisation of the nc scores is important for regression models [56, 57], to the best of our knowledge, it has not been applied to classification tasks so far. Including the KNN normalisation clearly improved validity for internal validation and the in-house dataset from 0.81 to 0.85 and from 0.59 to 0.82 at 0.2 SL, respectively (see Additional file 1: Table S2). Figure 3d,e show the lower error rate at a higher confidence area (small SLs), but decreased efficiency. Improved validity comes with the cost that less SCPs are made by the model, i.e. efficiency of 0.37 for ToxCast-AA and 0.21 for in-house-AA at 0.2 SL. From an application point of view, this is acceptable, since it is preferred to make no prediction rather than a wrong assertion.

Nevertheless, still, a high discrepancy between the accuracy of the active and inactive classes can be observed, with the highest discrepancy of 0.54 for the prediction of the external-AA data (see Additional file 1: Table S2). This is due to the high imbalance in the training data with a ratio of 1 active to 6.7 inactives in which the KNN algorithm is searching for nearest neighbours. While balancing in a mondrian ACP setting is normally not necessary, in the case of the additional KNN normalisation, random equal size sampling of the proper training and the calibration set, clearly reduced the discrepancy between the two classes for accuracy, as well as efficiency (see Table 5, Fig. 3g–i).

**Table 5:** Evaluation of normalised + balanced^a^ conformal prediction model for androgen receptor antagonism at 0.2 SL

Dataset	Purpose	Validity			Efficiency			Accuracy		
		All	cl.1^b^	cl.0^b^	All	cl.1^b^	cl.0^b^	All	cl.1^b^	cl.0^b^
ToxCast-AA	train model	0.85	0.84	0.85	0.57	0.39	0.60	0.89	0.76	0.91
In-house-AA	validation I	0.90	0.90	0.89	0.20	0.18	0.23	0.75	0.80	0.71
External-AA	validation II	0.80	0.76	0.82	0.43	0.33	0.52	0.74	0.67	0.78

^a^normalised nc score and balancing of calibration and proper training set

^b^cl.: class (class 1 = actives, class 0 = inactives)

The following factors should be noted regarding model performance: Firstly, the refined, normalised + balanced conformal predictors have been validated for use at low SLs. They are valid on the in-house dataset at SLs below 0.3, on the external dataset below 0.2. Therefore, predictions for the case study compounds are based on SL 0.2. As there is no interest in predictions with high error rates, the low validity at higher SLs can be ignored. Secondly, the three datasets all originate from different assays (i.e. performed in human, yeast, and hamster cells; a human androgen receptor was expressed in both human and yeast cells). Due to a limited amount of available toxicity data, it is inevitable to compare data from different organisms, nevertheless, caution should be exercised.

**Fig. 5:**
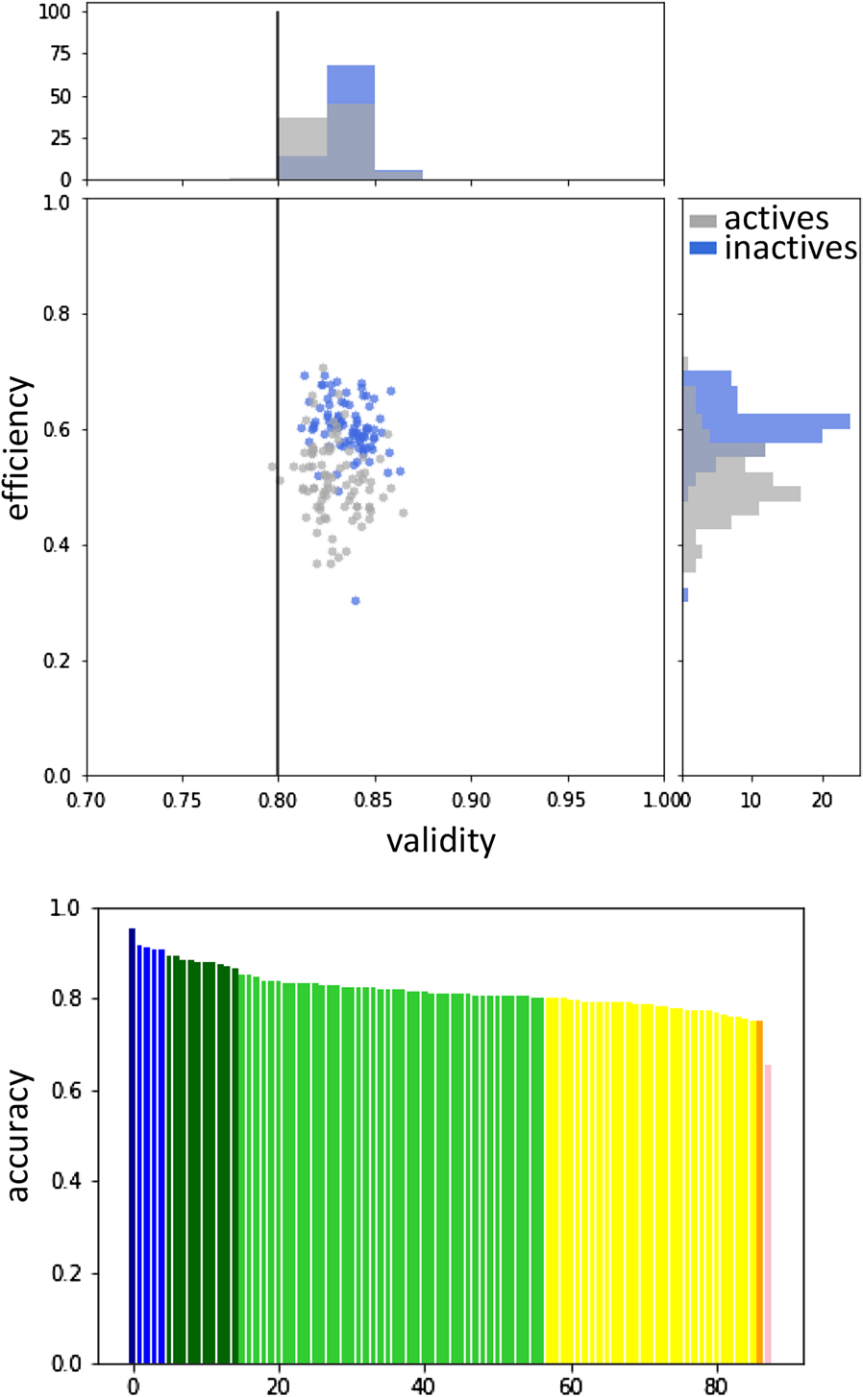
Evaluation of final 88 CP models. Top: validity vs. efficiency for inactives (blue) and actives (grey). Bottom: sorted, overall accuracy

The knowledge gained from creating the normalised + balanced model was applied to the remaining endpoints of the ToxCast dataset. Using the validated strategy, totally, 88 models were built with overall validity between 0.81 and 0.86, and overall efficiency between 0.32 and 0.68 at SL 0.2 (see Fig. 5, top). The accuracy of single class predictions ranged from 0.65 to 0.95 (see Fig. 5, bottom). Numbers for all 88 models and information about the endpoints can be found in Additional file 1: Tables S4 and S5, respectively.

### KnowTox—case study

**Fig. 6:**
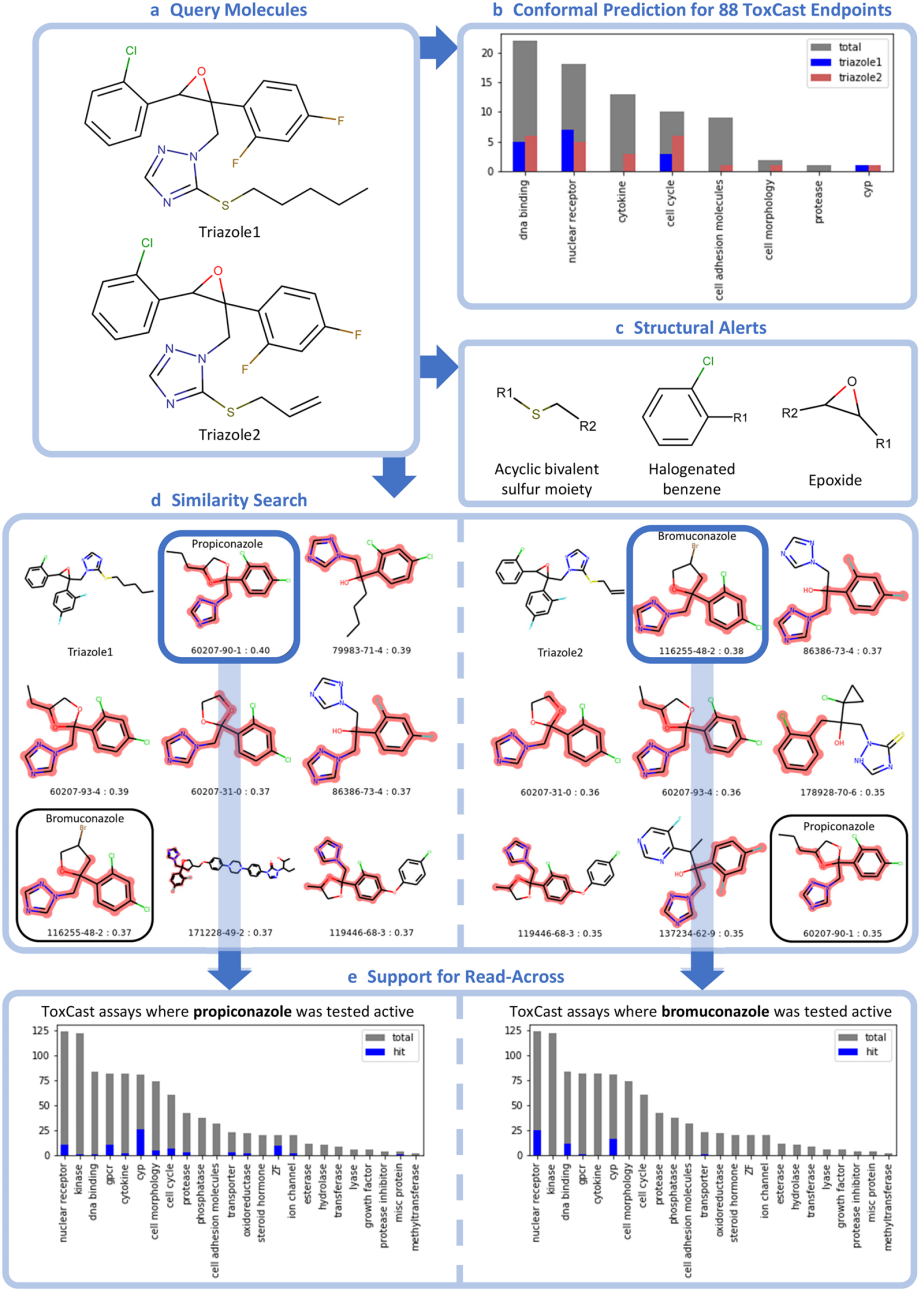
KnowTox tool applied in a case study. **a***Triazoles1&2* used as query compounds for the case study. **b** Output of CP. Grey: number of endpoints per family available for CP. Red and blue: number of endpoints where *triazoles1&2* were predicted to be active (SCP) at SL 0.2. **c** Three selected toxic alerts found for *triazoles1&2*. (Note that the potentially critical “triazole” substructure is not considered in this work). **d***Triazole1* (left) and *triazole2* (right) and their most similar molecules in ToxCast including CAS number and Tanimoto similarity. Red: maximum common substructure. **e** Experimental information from ToxCast for propiconazole (left) and bromuconazole (right). Grey: available assays in ToxCast. Blue: assays where compound was tested active

If KnowTox is queried with a compound of interest, three modules are envoked: conformal prediction (CP) for 88 endpoints, screening for unfavourable structural moieties, and support for read-across from similar compounds (see Fig. 6).

In this section, KnowTox usage is exemplified on two *triazoles* from the in-house dataset. They were designed as potential fungicides, but discontinued for various reasons. Both molecules share an epoxide structure with two halogenated phenyl moieties and a triazole ring with a thioether substitute (see Fig. 6a).

Firstly, a conformal prediction with every of the above described 88 models is made. Each model returns two p-values, one for the inactive (p0) and one for the active (p1) class. The higher p-value denotes the class the compound is most likely assigned to. For example, the ToxCast-AA model predicts *triazole1* to be active with p-values $\hbox {p}0=0.19$ and $\hbox {p}1=0.56$. In literature, CPs are often evaluated at a specified maximum accepted error rate (equivalent to SL $\epsilon$). For instance, if no more than 20% errors are accepted ($\hbox {SL} = 0.2$), the result is a prediction set containing all labels with p-values above 0.2. Thus, *triazole1* is predicted AA ({1}) while *triazole2* ($\hbox {p0}=0.21$, $\hbox {p1}=0.60$ for AA prediction) is assigned both labels ({0,1}). Therefore, no decision is made for *triazole2*. However, if 25% errors would be allowed ($\hbox {SL} = 0.25$), *triazole2* would also be predicted to be AA only ({1}).

Alternatively, evaluation can be independent from a predefined SL, i.e. with respect to credibility and confidence [28]. Credibility is defined as the largest p-value, this means the highest SL where a compound is still assigned to the corresponding label. Confidence is defined as 1–second largest p-value; since a high p-value of an alternate class reduces the confidence in the prediction. *Triazole1* is predicted to be AA with $\hbox {credibility}=0.56$ and $\hbox {confidence}=0.81$.

Referring to the three domains concept by Hanser et al. [27] (applicability, reliability, decidability), mentioned in the introduction, higher p-values, indicate higher reliability of a prediction while a large difference between the two p-values corresponds to increased decidability.

Considering the predictions by all 88 CP models (see Fig. 6b), both triazoles were predicted to be only active (SL 0.2) at a total of 15 endpoints, related to DNA binding, nuclear receptors, cell cycle as well as for aromatase inhibition (CYP19A1). A full list of the p-values for the predictions can be found in Additional file 1: Table S3.

Potential interaction of triazoles with aromatase can be explained through the mode of action of triazole fungicides. They inhibit the biosynthesis of ergosterol—an essential component of fungal cell membranes—changing the composition of the cell membrane. More precisely, the fungal enzyme lanosterol $14\alpha$-demethylase (CYP51) is inhibited which is closely related to human CYP15 and CYP19 (aromatase). Homology of fungal CYP51 to human CYP19 suggests likewise effects on steroidogenesis in humans [58]. Aromatase is responsible for catalysing the transformation of androgens into estrogens [59]. Inhibition can have a severe impact on hormone levels, though the actual physiological effects remain unclear [60, 61].

**Fig. 7:**
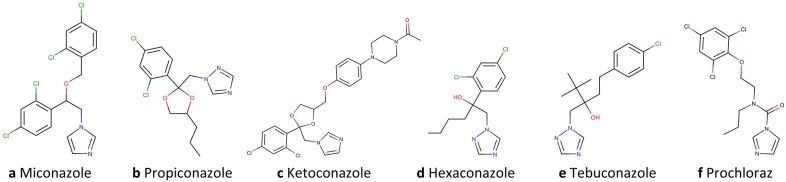
Chemical structures of triazole and imidazole fungicides referred to in the case study section

Besides, both *triazoles* were predicted to induce transcription factor activity and, thus, elevate the level of pregnane X receptor (PXR) response element and phenobarbital-responsive enhancer module mRNA. The two response elements are bound by members of the endogeneous human nuclear receptor subfamily 1 (PXR and constitutive androstane receptor (CAR), respectively), and are involved in overlapping pathways of xenobiotic detoxification, mainly occuring in the liver [62]. PXR is responsible for the expression of xenobiotic metabolising enzymes (e.g. cytochromes) in humans and is activated by a wide range of xenobiotics (e.g antibiotics) as well as endobiotics [63]. Activation of PXR has previously been observed by other azole fungicides such as miconazole and propiconazole [64] (see Fig. 7a, b). Moreoever, many conazoles are known to be involved in inhibition and induction of mammalian cytochromes P450 [65]. Generally, metabolism and elimination of foreign substances, such as fungicides, is favourable, it is mainly alarming when it comes to drug-drug interactions [66] (e.g. induction of xenobiotic metabolism by one drug may also affects metabolism and thus plasma levels of another drug). An example is the antimycotic drug ketoconazole (Fig. 7c) which is preferably applied topically rather than orally due to its high drug-drug interaction potential [67].

*Triazoles1&2* were both predicted to have antagonistic effects on the thyroid receptor. Indeed, thyroid endocrine effects of triazole fungizides have not yet extensively been studied: There is no indication of an effect in mammalians in vivo and only few reports in vitro and in zebrafish: Thyroid endocrine effects have previously been reported for two triazole fungicides hexaconazole and tebuconazole (see Fig. 7d, e) in zebrafish larvae [68]. Yu et al. suggested that the latter two triazoles can influence both, thyroid hormone levels and gene transcription in the hypothalamic-pituitary-thyroid axis. Changing thyroid hormone levels can affect several important physiological processes, e.g. tissue growth and differentiation, energy homeostasis, and metabolism [69, 70].

Furthermore, *triazoles1&2* were predicted to interfere with the cell cycle, i.e. leading to cytotoxicity. Also, in literature, evidence for cytotoxicity and cell cycle inhibition by triazole fungicides or mixtures containing such is given. For instance, Schwarzbacherova et al. reported cytotoxic and genotoxic effects, such as reduced cell viability, decreased cell proliferation, and apoptosis of bovine lymphocytes induced by fungicides [71]. In another study, they found bovine lymphocytes proliferation inhibited by a mixture of two conazole fungicides [72]. Additionally, Zhou et al. [73] described apoptotic effects of tebuconazole (see Fig. 7e) on human placental trophoblast cells.

Summarising, it could be shown that the CP models make reasonable predictions for potential toxic effects of these compounds, which could be substantiated with evidence in literature.

Secondly, with a search for structural alerts, toxicity prediction is supported with information from literature about substructures that have been previously assigned to specific toxic endpoints. Each query compound is screened against totally 919 available alerts and any critical substructure is highlighted.

Three alerts found for *triazoles1&2* are shown in Fig. 6c. According to Benigni et al. halogenated benzenes are prone to non-genotoxic carcinogenicity via agonistic or antagonistic interaction with the aryl hydrocarbon receptor  (AhR) [74]. AhR activation can result in altered gene expression and thus various types of toxicity, e.g. immunotoxicity, liver tumor promotion, and carcinogenicity [74, 75].

The sulfur moiety points to a study by Liu et al. [40], where 23 drugs containing acyclic bivalent sulfur moieties were investigated. Eight out of them are known for liver toxicity, another 14 are possibly hepatotoxic. Since only for one of the investigated drugs, liver toxicity could be excluded certainly, potential liver toxicity should be considered for these moieties. Conversely, this alert must not be an exclusion criterion, as the above drugs were still launched to the market.

Another warning is issued towards the epoxide substructure, a highly reactive group. Presence of the oxygen makes the carbons in the three-membered ring electrophilic. Thus they are typically accessed by nucleophiles, via an $S_N2$-type mechanism resulting in ring opening and a covalent bond. This may cause mutagenic or carcinogenic effects, as well as skin sensitization and aquatic toxicity [39, 76–78]. While the nucleophile preferentially attacks the less substituted ring carbon, [76] in the case of *triazoles1&2*, access to any ring carbon is sterically hindered due to the three surrounding substituents. Thus, the present epoxides can be considered inert.

Note that the issued warnings are based on the 919 toxic alerts incorporated into KnowTox. If the collection of structural alerts is desired to be even more comprehensive, it can always be extended by literature or in-house knowledge. For example, the triazole substructure, which is also included in the ToxAlerts tool as an “extended functional group” [19, 79], is not considered in this work. As seen in the CP part, this moiety is responsible for both, the antifungal activity, and adverse effects due to aromatase inhibition.

Thirdly, risk assessment is complemented through inclusion of information from experimental ToxCast assay outcomes of similar molecules. For a query compound, the 7912 compounds of the ToxCast dataset are screened to identify the molecules with highest Tanimoto similarity and toxicity information of these most similar molecules is displayed. To simplify the assessment of the grade of similarity, and thus the reliability in the read-across, the Tanimoto index, as well as the MCS between the molecules are indicated. Similarity search and support for read-across can especially be valuable for those endpoints where minority class data was too few to build a CP model.

When querying the triazoles in the similarity search, eight fairly similar molecules are returned (see Fig. 6d). The similarity is mainly reflected in the triazole substructure and halogenated benzenes, mostly connected in three- or four-membered ring-systems. Note that no other molecule with an epoxide substructure is captured within the similarity search.

Assuming that the found molecules are similar enough, known experimental information about them could be used to support read-across. Although ToxCast provides data from 985 assays, the most similar molecules to the two triazoles were only assayed for 32 to 639 endpoints each. The most similar molecule to *triazole1*, propiconazole, was, amongst others, tested active at several nuclear receptor-related endpoints (e.g. PXR*e*, CAR, androgen, thyroid and estrogen receptors), cytochromes P450 (i.e. 19, 1a, 2b, 2c, 2d, 3a), and GPCRs (e.g. opioid receptors, muscarinic cholinergic receptors, and histamine receptor H2). Furthermore, it had effects on several developmental endpoints of zebrafish embryos [80] (see Fig. 6e). Experimentally observed activity for bromuconazole, the most similar compound to *triazole2*, was mainly restricted to nuclear receptors (e.g. retinoic acid, androgen, and estrogen receptors, PXR), DNA binding (AhR, p53, sterol regulatory element binding protein), and cytochromes P450 (19A1, 2a, 2b, 2c, 3a). It should, moreover, be noted that Br-substituents, as in bromuconazole, are generally more reactive than F- or Cl-substituents [39]. So, certain toxic effects might be more distinct in bromuconazole than in molecules without Br- substituted moieties, such as *triazoles1&2*.

The toxic effects described for *triazoles1&2* above can be related to pathways, such as CAR/RXR and PXR/RXR activation, xenobiotic metabolism signaling, and AhR signaling, which were also investigated in a study by Hester et al. [65] and related to hepatocarcinogenesis.

An association of bromuconazole with xenobiotic metabolism and nuclear receptors (i.e. PXR), as suggested by the similarity-based read-across, is further supported by a recent study by Abdelhadya et al. [81]. They reported, inter alia, that the liver oxidative damage is associated with increased PXR activity and concurrent decrease in expression of the CAR gene.

In conclusion, indications of liver toxicity, liver enzyme induction, and aromatase inhibition were found in rats treated with these two triazoles in in-house studies. Thus, further development of these two triazole candidates was discontinued. Also, according to literature, several conazole fungicides have been associated to potential AA endocrine disruption [82]. For example, AA effects were reported for prochloraz (Fig. 7f) in human prostate cancer cells [83]. Also, propiconazole (Fig. 7b) showed AA activity in vitro, though it could not be asserted in vivo [84]. Moreover, another explanation for triazole-induced liver toxicity was recently provided by Knebel et al. who investigated molecular mechanisms of hepatic steatosis [85]. The triazole fungicides propiconazole and tebuconazole (see Fig. 7b, e) were shown to influence the expression of steatosis-related genes. Especially, the observed additivity of equimolar mixtures suggests a common mode of action.

To conclude, KnowTox was able to predict many interactions, especially with respect to the induction of xenobiotic enzymes, endocrine effects, and liver toxicity. The discussed predictions could be supported by literature findings for other related molecules. Also, the KnowTox tool could reproduce the main in vivo effects of two *triazole* compounds, which have been discontinued as development candidates.

To sum up, such a holistic analysis of the toxic potential of a novel molecule can be of high reward in compound (de-)selection, planning further toxicity testing, and to support read-across. Nevertheless, its benefit can still be increased by incorporation of larger datasets, biological activity fingerprints characterising the compounds, and in vivo endpoint data for model development. Note that KnowTox is based on the ToxCast dataset chosen for its size, scope and accessibility. Used in early stages of new chemical’s development, the tool can provide a broad overview on possible interactions with toxicity-related targets. For application in regulatory toxicity testing, it is beneficial to have toxicity data which fundamentally support regulatory required toxicity assays in animals, e.g. reproduction toxicity studies. In case of occurrence of toxic effects, the tool will help to identify a potential mode-of-action. In addition, it will increase certainty if data support the absence of toxic effects. Thus, if, in future, sufficient standard toxicity data will be available for model training, the introduced pipeline has the potential to become even more powerful. Also, information about the compound’s bioavailability and in vitro to in vivo translation of the assays would be of high interest [10, 86–88]. According to Grenet et al. [87], it seems to be more challenging to predict long-term in vivo endocrine disruption, compared to predicting short-term in vivo endocrine effects. Furthermore, for a complete risk assessment, the quantitative dose-response needs to be considered. That is beyond the scope of this paper. Information on the type and amount of formed metabolites is highly desirable (see the prominent role of xenobiotic metabolism in the toxic effects of triazole fungicides).

In vitro toxicology has embarked on combining data from different sources to derive more reliable and more relevant information on potential toxic effects of compounds [89, 90]. This concept also applies to in silico toxicology and combinations of the different in vivo, in vitro, in silico methods: combining the input from different, complementary models can provide advantageous information which cannot be obtained from one single source.

## Conclusion

In silico methods for toxicity prediction are promising tools assisting in the reduction and replacement of animal testing. In this work, three different approaches were combined in order to support holistic risk assessment for new query molecules.

In praxis, it is not only important to have well performing models, but also to know that they can be confidently applied to novel compounds (applicability domain), that the predictions are reliable (reliability domain) and informative (decidability domain). A popular technique for confidence estimation for machine learning models is conformal prediction, which enables straightforward training of valid and balanced models with little optimisation effort. While this advantage was also witnessed during internal validation, in this work, some challenges emerged during application to an external dataset where exchangeability was not given. Therefore, the models were refined in two steps: firstly, using k-nearest neighbour normalisation improved validity of both internal and in-house data predictions (reliability domain). Secondly, random equal size sampling of the training set improved informational efficiency of the predictions (decidability domain). This strategy was initially validated on an AA model and subsequently transferred to totally 88 ToxCast endpoint models. Complemented with structural alerts from literature and providing support for read-across, the KnowTox tool generates a risk assessment picture to examine potential toxicity of a novel query compound from different angles as exemplified by the case study on two triazoles.

## Supplementary information

**Additional file 1.** Additional Tables S1–S5.

## Data Availability

ToxCast data was used for model training and is publicly available at https://figshare.com/articles/ToxCast_and_Tox21_Data_Spreadsheet/6062503 [41]. For in depth evaluation and applicability optimization of the ToxCast-AA model the external-AA and in-house-AA datasets were used. External-AA data are available from Norinder et al. [33]. The 534 in-house-AA data are proprietary to BASF SE. The structures of the two BASF SE case study molecules are shown in this manuscript. The pre-processed ToxCast and External-AA data, as well as a notebook demonstrating the process of training and evaluating conformal prediction models, based on this manuscript’s methods, is available under https://github.com/volkamerlab/knowtox_manuscript_SI.

## Abbreviations

ML: Machine learning
MCS: Maximum common substructure
QSAR: Quantitative structure-activity relationship
PCA: Principal component analysis
ACP: Aggregated conformal predictor
SL: Significance level
CP: Conformal prediction
nc score: Nonconformity score
AA: Androgen receptor antagonism
SCP: Single class prediction
PXR: Pregnane X receptor
AhR: Aryl hydrocarbon receptor
CAR: Constitutive androstane receptor
