# Supplementary material for: KnowTox: pipeline and case study for confident prediction of potential toxic effects of compounds in early phases of development
Source: J Cheminform. 2020 Apr 14;12:24. doi: 10.1186/s13321-020-00422-x (PMC7157991; doi:10.1186/s13321-020-00422-x)
Supplement: Supplementary file 1 — Additional file 1. Additional Tables S1–S5. [file 13321_2020_422_MOESM1_ESM.pdf]

Additional file

KnowTox: Pipeline and Case Study for Confident Prediction of Potential Toxic Effects of Compounds in Early Phases of Development

Andrea Morger, Miriam Mathea, Janosch H Achenbach, Antje Wolf, Roland Buesen, Klaus-Juergen Schleifer, Robert Landsiedel and Andrea Volkamer

Table S1: Evaluation of original conformal prediction model for androgen receptor antagonism at 0.2 significance level (SL)

		Validity			Efficiency			Accuracy		
Dataset	Purpose	all	cl.1[a]	cl.0[a]	all	cl.1	cl.0	all	cl.1	cl.0
ToxCast-AA	train model	0.81	0.82	0.81	0.87	0.89	0.87	0.78	0.80	0.78
In-house-AA	validation I	0.59	0.98	0.16	0.94	0.98	0.91	0.56	0.97	0.07
External-AA	validation II	0.75	0.77	0.74	0.79	0.77	0.81	0.68	0.70	0.67

[a]cl.: class (class 1 = actives, class 0 = inactives)

Table S2: Evaluation of normalised conformal prediction model (normalised nonconformity score) for androgen receptor antagonism at 0.2 SL

		Validity			Efficiency			Accuracy		
Dataset	Purpose	all	cl.1[a]	cl.0[a]	all	cl.1	cl.0	all	cl.1	cl.0
ToxCast-AA	train model	0.85	0.82	0.85	0.37	0.14	0.40	0.95	0.46	0.98
In-house-AA	validation I	0.82	0.84	0.80	0.21	0.25	0.16	0.85	0.94	0.71
External-AA	validation II	0.75	0.65	0.83	0.29	0.19	0.37	0.77	0.39	0.93

[a]cl.: class (class 1 = actives, class 0 = inactives)

	Triazole1		Triazole2	
**Endpoint**	**p0**	**p1**	**p0**	**p1**
1	0.140	0.747	0.123	0.572
45	0.079	0.522	0.086	0.367
63	0.405	0.457	0.466	0.566
64	0.193	0.444	0.169	0.397
66	0.154	0.424	0.142	0.407
69	0.214	0.623	0.241	0.720
74	0.294	0.700	0.226	0.686
75	0.342	0.411	0.220	0.334
82	0.391	0.676	0.381	0.680
84	0.425	0.734	0.364	0.739
91	0.187	0.597	0.134	0.517
97	0.200	0.648	0.186	0.648
98	0.323	0.724	0.289	0.714
100	0.386	0.804	0.319	0.744
101	0.142	0.571	0.178	0.623
102	0.393	0.471	0.268	0.429
103	0.013	0.281	0.024	0.436
104	0.236	0.615	0.221	0.570
106	0.433	0.652	0.230	0.569
107	0.056	0.459	0.108	0.625
113	0.073	0.379	0.082	0.445
114	0.300	0.668	0.248	0.690
117	0.444	0.328	0.270	0.299
134	0.395	0.615	0.334	0.566
135	0.307	0.633	0.202	0.448
142	0.630	0.264	0.493	0.181
145	0.399	0.609	0.273	0.364
147	0.386	0.765	0.285	0.671
153	0.411	0.711	0.221	0.398
157	0.287	0.808	0.218	0.675
159	0.373	0.761	0.256	0.593
165	0.417	0.787	0.284	0.575
167	0.368	0.470	0.299	0.238
169	0.357	0.708	0.172	0.369
171	0.452	0.563	0.442	0.422
173	0.388	0.774	0.322	0.655
175	0.478	0.616	0.304	0.414
177	0.426	0.798	0.253	0.534
179	0.350	0.807	0.213	0.590
181	0.391	0.691	0.320	0.557
185	0.326	0.762	0.303	0.713
189	0.408	0.662	0.356	0.599
221	0.329	0.811	0.173	0.599
235	0.400	0.663	0.249	0.376
241	0.441	0.631	0.276	0.373
243	0.431	0.627	0.300	0.498
249	0.439	0.700	0.240	0.440
251	0.092	0.559	0.071	0.461
253	0.419	0.571	0.274	0.263
257	0.549	0.509	0.302	0.275
267	0.416	0.658	0.261	0.483
277	0.257	0.692	0.176	0.554
287	0.373	0.721	0.212	0.467
291	0.383	0.797	0.271	0.483
297	0.351	0.749	0.268	0.480
299	0.468	0.733	0.322	0.527
301	0.444	0.714	0.308	0.571
303	0.378	0.819	0.166	0.584
305	0.347	0.799	0.192	0.593
307	0.466	0.810	0.262	0.635
309	0.358	0.745	0.185	0.517
315	0.299	0.866	0.165	0.777
317	0.356	0.723	0.177	0.535
762	0.191	0.558	0.214	0.597
765	0.205	0.640	0.213	0.622
767	0.036	0.423	0.086	0.558
785	0.408	0.357	0.410	0.353
786	0.283	0.690	0.244	0.601
788	0.602	0.153	0.648	0.259
789	0.205	0.681	0.232	0.600
793	0.171	0.519	0.227	0.480
794	0.399	0.580	0.337	0.585
797	0.498	0.612	0.389	0.488
804	0.193	0.705	0.190	0.703
806	0.554	0.338	0.593	0.480
1110	0.262	0.692	0.232	0.709
1113	0.067	0.322	0.330	0.662
1116	0.325	0.594	0.269	0.477
1120	0.247	0.767	0.255	0.684
1127	0.289	0.636	0.339	0.590
1317	0.212	0.642	0.174	0.670
1321	0.342	0.593	0.406	0.678
1325	0.242	0.615	0.216	0.498
1329	0.264	0.547	0.222	0.440
1412	0.183	0.558	0.197	0.576
1441	0.297	0.782	0.244	0.695
1496	0.278	0.844	0.277	0.795
1816	0.159	0.436	0.194	0.464

Table S3: P-values obtained from predictions with 88 KnowTox conformal prediction models for *triazoles1&2*.

	Accuracy		Validity		Efficiency	
**Endpoint**	**cl0**	**cl1**	**cl0**	**cl1**	**cl0**	**cl1**
1	0.82	0.76	0.84	0.82	0.59	0.56
45	0.84	0.72	0.85	0.81	0.57	0.56
63	0.84	0.78	0.85	0.83	0.62	0.61
64	0.83	0.74	0.84	0.83	0.59	0.5
66	0.83	0.77	0.83	0.83	0.62	0.47
69	0.79	0.72	0.83	0.84	0.61	0.44
74	0.81	0.76	0.81	0.82	0.69	0.49
75	0.74	0.79	0.83	0.83	0.49	0.63
82	0.76	0.7	0.83	0.82	0.57	0.52
84	0.81	0.76	0.83	0.83	0.63	0.53
91	0.85	0.78	0.84	0.84	0.66	0.52
97	0.83	0.76	0.82	0.82	0.64	0.52
98	0.84	0.72	0.84	0.82	0.61	0.47
100	0.81	0.77	0.83	0.83	0.66	0.54
101	0.85	0.79	0.83	0.82	0.65	0.45
102	0.85	0.71	0.84	0.83	0.67	0.5
103	0.82	0.8	0.82	0.82	0.65	0.66
104	0.85	0.77	0.84	0.84	0.64	0.45
106	0.83	0.62	0.86	0.83	0.53	0.39
107	0.84	0.8	0.82	0.83	0.68	0.58
113	0.84	0.81	0.82	0.83	0.68	0.61
114	0.83	0.72	0.84	0.84	0.59	0.47
117	0.78	0.81	0.81	0.82	0.6	0.71
134	0.87	0.77	0.83	0.83	0.65	0.5
135	0.85	0.79	0.82	0.82	0.69	0.65
142	0.73	0.81	0.82	0.83	0.52	0.66
145	0.8	0.71	0.84	0.8	0.56	0.51
147	0.81	0.76	0.85	0.84	0.64	0.49
153	0.82	0.8	0.83	0.85	0.6	0.47
157	0.8	0.77	0.83	0.82	0.62	0.56
159	0.82	0.79	0.82	0.82	0.59	0.45
165	0.83	0.78	0.84	0.84	0.58	0.43
167	0.77	0.77	0.83	0.84	0.52	0.52
169	0.81	0.78	0.84	0.84	0.56	0.46
171	0.81	0.72	0.84	0.83	0.59	0.5
173	0.84	0.8	0.84	0.81	0.62	0.5
175	0.84	0.79	0.85	0.85	0.59	0.49
177	0.81	0.78	0.85	0.85	0.59	0.46
179	0.8	0.75	0.85	0.82	0.58	0.5
181	0.81	0.75	0.83	0.82	0.61	0.56
185	0.81	0.75	0.83	0.81	0.58	0.49
189	0.83	0.77	0.85	0.85	0.6	0.48
221	0.78	0.76	0.83	0.82	0.57	0.56
235	0.81	0.8	0.83	0.84	0.65	0.56

	Accuracy		Validity		Efficiency	
**Endpoint**	**cl0**	**cl1**	**cl0**	**cl1**	**cl0**	**cl1**
241	0.78	0.74	0.84	0.84	0.54	0.51
243	0.83	0.75	0.84	0.83	0.62	0.57
249	0.82	0.75	0.84	0.83	0.59	0.52
251	0.78	0.81	0.84	0.85	0.59	0.55
253	0.81	0.77	0.82	0.82	0.6	0.54
257	0.82	0.75	0.86	0.82	0.53	0.48
267	0.81	0.76	0.85	0.8	0.56	0.53
277	0.77	0.81	0.82	0.86	0.6	0.59
287	0.81	0.76	0.85	0.82	0.6	0.49
291	0.84	0.77	0.84	0.82	0.57	0.44
297	0.76	0.75	0.82	0.85	0.58	0.46
299	0.81	0.8	0.83	0.84	0.62	0.48
301	0.8	0.75	0.83	0.82	0.61	0.48
303	0.78	0.74	0.83	0.84	0.61	0.51
305	0.77	0.74	0.83	0.83	0.61	0.52
307	0.83	0.8	0.83	0.85	0.61	0.51
309	0.8	0.76	0.84	0.86	0.57	0.5
315	0.78	0.77	0.82	0.83	0.61	0.59
317	0.82	0.8	0.83	0.84	0.61	0.51
762	0.91	0.76	0.85	0.84	0.6	0.39
765	0.9	0.77	0.84	0.83	0.57	0.38
767	0.88	0.77	0.84	0.82	0.6	0.47
785	0.88	0.85	0.84	0.81	0.58	0.62
786	0.93	0.76	0.85	0.83	0.6	0.37
788	0.61	0.85	0.84	0.86	0.3	0.45
789	0.92	0.79	0.83	0.83	0.6	0.41
793	0.87	0.84	0.83	0.81	0.66	0.54
794	0.83	0.84	0.85	0.84	0.57	0.55
797	0.97	0.77	0.85	0.82	0.65	0.37
804	0.9	0.81	0.83	0.81	0.64	0.53
806	0.91	0.85	0.86	0.83	0.67	0.59
1110	0.82	0.78	0.83	0.83	0.61	0.57
1113	0.79	0.79	0.83	0.82	0.57	0.54
1116	0.89	0.78	0.84	0.82	0.59	0.46
1120	0.88	0.76	0.83	0.82	0.68	0.5
1127	0.89	0.66	0.85	0.82	0.66	0.42
1317	0.81	0.78	0.84	0.83	0.59	0.56
1321	0.81	0.77	0.84	0.82	0.54	0.53
1325	0.89	0.72	0.86	0.81	0.56	0.45
1329	0.86	0.76	0.85	0.85	0.54	0.44
1412	0.83	0.74	0.83	0.82	0.68	0.57
1441	0.79	0.67	0.84	0.81	0.58	0.5
1496	0.76	0.73	0.82	0.82	0.61	0.57
1816	0.92	0.81	0.84	0.84	0.68	0.48

Table S4: Class-wise evaluation of conformal prediction models.

Endpoint_ID	Description	Endpoint Family	Endpoint Subfamily	# Actives	# Inactives
1	T47D	cell cycle	cytotoxicity	413	1169
45	CellLoss	cell cycle	cytotoxicity	396	533
63	Ahr	dna binding	basic helix-loop-helix protein	397	2670
64	AP	dna binding	basic leucine zipper	557	2501
66	BRE	dna binding	Smad protein	349	2721
69	CRE	dna binding	basic leucine zipper	303	2760
74	EGR	dna binding	zinc finger	441	2624
75	ERE	nuclear receptor	steroidal	794	2254
82	HIF1a	dna binding	basic helix-loop-helix protein	335	2731
84	HSE	dna binding	heat shock protein	369	2696
91	MRE	dna binding	zinc finger	647	2417
97	NRF2	dna binding	basic leucine zipper	1164	1878
98	Oct	dna binding	POU domain protein	481	2578
100	Pax6	dna binding	paired box protein	405	2664
101	PBREM	nuclear receptor	non-steroidal	346	2721
102	PPRE	nuclear receptor	non-steroidal	469	2591
103	PXRE	nuclear receptor	non-steroidal	1494	1549
104	RORE	nuclear receptor	orphan	333	2739
106	Sp1	dna binding	zinc finger	302	2760
107	SREBP	dna binding	basic helix-loop-helix leucine zipper	473	2591
113	VDRE	nuclear receptor	non-steroidal	819	2231
114	Xbp1	dna binding	basic leucine zipper	401	2662
117	ERa	nuclear receptor	steroidal	678	2388
134	PPARg	nuclear receptor	non-steroidal	852	2206
135	PXR	nuclear receptor	non-steroidal	873	2177
142	RXRb	nuclear receptor	non-steroidal	479	2580
145	Eselectin	cell adhesion molecules	selectins	376	947
147	HLADR	cell adhesion molecules	MHC Class II	510	812
153	MCP1	cytokine	chemotactic factor	346	976
157	Proliferation	cell cycle	cytotoxicity	554	767
159	SRB	cell cycle	cytotoxicity	433	886
165	uPAR	cytokine	plasmogen activator	382	940
167	VCAM1	cell adhesion molecules	Immunoglobulin CAM	328	997
169	Vis	cell morphology	cell conformation	396	921
171	Eotaxin3	cytokine	chemotactic factor	403	920
173	MCP1	cytokine	chemotactic factor	358	964
175	Pselectin	cell adhesion molecules	selectins	372	954
177	SRB	cell cycle	cytotoxicity	361	962
179	uPAR	cytokine	plasmogen activator	319	1005
181	VCAM1	cell adhesion molecules	Immunoglobulin CAM	400	923
185	HLADR	cell adhesion molecules	MHC Class II	356	965
189	IP10	cytokine	chemotactic factor	349	974
221	Proliferation	cell cycle	cytotoxicity	401	920
235	CollagenIII	cell adhesion molecules	collagen	403	915
241	IP10	cytokine	chemotactic factor	384	933
243	MCSF	cytokine	colony stimulating factor	388	932
249	PAI1	cytokine	plasmogen activator inhibitor	367	957
251	Proliferation	cell cycle	cytotoxicity	575	746
253	SRB	cell cycle	cytotoxicity	312	1012
257	VCAM1	cell adhesion molecules	Immunoglobulin CAM	383	939
267	MMP9	protease	matrix metalloproteinase	359	965
277	CD40	cytokine	inflammatory factor	397	927
287	MCSF	cytokine	colony stimulating factor	358	962
291	SRB	cell cycle	cytotoxicity	341	981
297	VCAM1	cell adhesion molecules	Immunoglobulin CAM	405	919
299	CD38	cytokine	other cytokine	404	918
301	CD40	cytokine	inflammatory factor	399	922
303	CD69	cytokine	inflammatory factor	370	953
305	Eselectin	cell adhesion molecules	selectins	377	945
307	IL8	cytokine	interleukins	315	1005
309	MCP1	cytokine	chemotactic factor	318	1004
315	Proliferation	cell cycle	cytotoxicity	522	802
317	SRB	cell cycle	cytotoxicity	333	990
762	AR	nuclear receptor	steroidal	868	5845
765	AR	nuclear receptor	steroidal	603	6161
767	Aromatase	cyp	steroidogenesis-related	925	5770
785	ERa	nuclear receptor	steroidal	323	6462
786	ERa	nuclear receptor	steroidal	761	5969
788	ERa	nuclear receptor	steroidal	857	5716
789	ERa	nuclear receptor	steroidal	494	6265
793	GR	nuclear receptor	steroidal	369	6431
794	GR	nuclear receptor	steroidal	333	6471
797	MMP	cell morphology	organelle conformation	705	3810
804	TR	nuclear receptor	non-steroidal	1390	5345
806	AhR	dna binding	basic helix-loop-helix protein	641	6106
1110	ARE	dna binding	basic leucine zipper	1199	4871
1113	HSE	dna binding	heat shock protein	404	5781
1116	p53	dna binding	tumor suppressor	593	6167
1120	FXR	nuclear receptor	non-steroidal	698	5467
1127	PPARg	nuclear receptor	non-steroidal	389	5813
1317	p53	dna binding	tumor suppressor	735	5975
1321	p53	dna binding	tumor suppressor	663	6033
1325	p53	dna binding	tumor suppressor	668	6048
1329	p53	dna binding	tumor suppressor	648	6082
1412	DR4	nuclear receptor	non-steroidal	624	2438
1441	ISRE	dna binding	interferon regulatory factors	405	2655
1496	TCF	dna binding	HMG box protein	384	2670
1816	AR	nuclear receptor	steroidal	916	5286

Table S5: Information about endpoints where conformal prediction models are available. More details about all endpoints can be found under: https://figshare.com/articles/ToxCast_and_Tox21_Data_Spreadsheet/6062503.
